# Cerebro-Nervous Disorders Peculiar to Women

**Published:** 1883-12

**Authors:** Thomas Madden Moore

**Affiliations:** Dublin


### CEREBRO-NERVOUS DISORDERS PECULIAR TO WOMEN.

THOS. MADDEN MOORE, M. D., DUBLIN.

Dr. Moore contributes a paper on the Cerebro-Nervous Disorders Peculiar to Women in the November number of the *American Journal of Obstetrics*, etc., from which we take the following abstracts:

An increasing tendency to nervous and mental disorders, especially among women, has become manifest in the past few years. Thus, in the comparatively short period covered by the annual reports of the English Commissioners in Lunacy, the number of the insane have more than doubled, having increased from one in eight hundred to one in three hundred and fifty of the population. Formerly lunacy was more prevalent among men; now there are more insane women than men in this country. There is a similar increase of nervous disorders generally, and of hysteria in particular, etiologically connected, I believe, with certain utero-ovarian disorders that have recently come into prominence.

I desire now to submit a few observations, mainly the result of clinical experience, on the relations between certain utero-ovarian disorders, and other morbid conditions peculiar to women, and the most frequent type of cerebro-nervous diseases, as exemplified by the protean forms of hysteria and the various degrees of mental derangement connected with pregnancy, parturition, and lactation.

The predominant influence of the reproductive system is manifest in women, in every vital action from the approach of puberty until the menopause. Throughout this long period, at every menstrual epoch there may, almost invariably, be observed as coincident occurrences of constitutional and nervous disturbance. When menstruation has become fairly established and is normal in every respect, then this disturbance may be so slight as to escape notice. But the earlier catamenial epochs, and every subsequent deviation from healthy menstruation, as well as the climacteric period, are, as a rule, accompanied by some manifestation of hysteria.

The functional connection between the cerebro-nervous and reproductive systems is apparent in nearly all chronic uterine and ovarian complaints. Patients lose flesh, suffer from cardialgia, dyspepsia, palpitation, intense headache, become cachetic-looking, despondent, anxious, excitable, or irritable to the verge of insanity. In other words, as the uterine disease progresses, its local evidences become obscured by its constitutional and nervous consequences. Foremost among these, hysteria in every variety, from the ordinary hysterical paroxysm, which is too generally wrongly overlooked, to the gravest forms of cerebro-nervous disorders, namely, epilepsy and insanity, which may result from neglect of warnings thus furnished.

Among the most important mental disorders and nervous affections arising from uterine and ovarian troubles are:

I. Lunacy in its various forms. Of eighty-five thousand one hundred and sixty-seven registered lunatics in Great Britain, forty-eight thousand five hundred and eighty-six are females. Among the causes of this increase of female insanity, besides uterine and ovarian disorders, must be reckoned the assumption of masculine privileges and modes of life, seeking higher education and attempting to do man's work.

The etiological connection between catamenial derangements, especially suppression of menses, and mental disturbance has long been recognized. Amenorrheal insanity was described as far back as the time of Pinel, who relates, among others, the case of a girl who was in a state of dementia, which continued for some years, during which she never menstruated. One day, however, she suddenly ran to her mother, exclaiming, "I am well." The catamenia had just flowed, and her reason was immediately restored.

II. The mental and nervous disorders of pregnancy and the puerperal state are well known to all physicians. The irritable mind, the depraved appetite and morbid fancies, also puerperal mania, are most common exhibitions of this kind. The uterine origin of certain forms of mental disorder is strikingly evidenced in puerperal mania or insanity consequent on parturition. This is usually preceded by the premature suppression or diminution of the lochia, which becomes fetid as well as scanty in such cases.

III. Hysterical Pseudocyesis or feigned pregnancy is a curious mental condition associated with these disorders. Spurious pregnancy is invariably connected with functional disorders of the utero-ovarian system and usually occurs about the time of the final cessation of the menses, although more than once I have been consulted in cases of spurious pregnancy occurring in women under twenty years of age. In many cases, I have found the symptoms of spurious pregnancy hardly distinguishable from those of true pregnancy. Thus we often meet with cases of complete amenorrhea, followed by morning sickness, turgescence of the mammæ, and enlargement of abdomen, occurring in middle-aged hysterical married women who desire to be thought pregnant. Sterile women under such circumstances not uncommonly become hysterically insane on this subject and take extraordinary, and often successful, pains to persuade those about them, as well as themselves, that they are "as ladies who love their lords should be," when only suffering from the change of life, dyspepsia, dropsy, or mere obesity.

IV. Every variety of epileptiform disease in women is most frequent in those of a hysterical temperament and is generally found associated with amenorrhea or some other disorder of the sexual system.

V. Hysterical trances occur from menstrual or uterine disorders. "One of the most curious forms of hysteria," says Dr. Elliotson, "is long-continued insensibility, and is called a trance. Sometimes there is insensibility for a few days, and sometimes for many weeks." This insensibility is so profound as to counterfeit death, and patients have actually been consigned to the tomb, or only rescued from it by some happy accident.

VI. Hysterical paralysis is another form of reflect functional nervous disorder which is in some cases traceable to utero-ovarian causes.

We may find every degree of loss of voluntary power, from the most trivial local weakness to complete paraplegia.

Some reference is necessary in this connection to the increasing prevalence of *alcoholism* in all classes of modern society, among women as well as men. Moreover, to the utero-ovarian disorders which have been alluded to as connected with nervous and mental complaints may in some instances be also ascribed a place in the causation of intemperance in women. I have repeatedly traced the craving for alcohol in confirmed inebriates to the first painful menstrual period, when stimulants are too commonly pressed by foolish advisers. The pains of dysmenorrhea once thus relieved, at the next epoch the patient naturally, and no longer unwillingly, seeks similar solace, and thus "this unkind Nepenthe" is gradually employed in increasing doses, until at last the victim of dysmenorrheal alcoholism becomes—perhaps unconsciously—an habitual, and too often an incurable, drunkard.

---

The above condensation gives the main points of Dr. Moore's interesting paper. What does it teach us as homœopathists?—To diagnose and treat a disease? To find the *cause* of the disease and remove it? This latter would be of service, could we do it; but we (homœopathists) must remember that we have, in such cases as these, something more than a local disease to treat. Hahnemann has pointed out, and subsequent experience has proved, that there are constitutional causes back of these local disorders which must be eradicated before the local disease is cured.

There can be no doubt that the worst cases of headache, and that nervous disorders of all kinds, have their origin in the local maltreatment of these uterine diseases. A leucorrheal discharge is suppressed by local applications, ulcers are cauterized, and, as a consequence, the disease, driven from the uterus, appears elsewhere in a worse form. Sterility, besides a multitude of aches and pains, is one of the *boons* conferred on womankind by the modern "science" of gynæcology. E. J. L.

